# Serum amyloid A, a host-derived DAMP in pregnancy?

**DOI:** 10.3389/fimmu.2022.978929

**Published:** 2022-08-05

**Authors:** Yi-kai Lin, Ping Zhu, Wang-sheng Wang, Kang Sun

**Affiliations:** ^1^ Center for Reproductive Medicine, Ren Ji Hospital, School of Medicine, Shanghai Jiao Tong University, Shanghai, China; ^2^ Shanghai Key Laboratory for Assisted Reproduction and Reproductive Genetics, Shanghai, China; ^3^ Department of Obstetrics and Gynecology, No.971 Hospital of the PLA Navy, Qingdao, China

**Keywords:** acute phase protein, inflammation, parturition, pregnancy, SAA, DAMP, gestation

## Abstract

Serum amyloid A (SAA) is one of the acute phase proteins released primarily from the liver in response to infection, inflammation and trauma. Emerging evidence indicates that SAA may function as a host-derived damage-associated molecular pattern (DAMP) protein to sense danger signals in pregnancy. The plasma SAA levels in maternal circulation are significantly increased in normal parturition, particularly in postpartum, as well as in gestational disorders such as premature preterm rupture of membranes, pre-eclampsia, gestational diabetes, and recurrent spontaneous abortion. It is likely that SAA acts as a non-specific DAMP molecule in response to inflammation and trauma experienced under these conditions. Notably, SAA can also be synthesized locally in virtually all gestational tissues. Within these gestational tissues, under the induction by bacterial products, pro-inflammatory cytokines and stress hormone glucocorticoids, SAA may exert tissue-specific effects as a toll-like receptor 4 (TLR4)-sensed DAMP molecule. SAA may promote parturition through stimulation of inflammatory reactions *via* induction of pro-inflammatory cytokines, chemokines, adhesion molecules and prostaglandins in the uterus, fetal membranes and placenta. In the fetal membranes, SAA may also facilitate membrane rupture through induction of matrix metalloproteases (MMPs)- and autophagy-mediated collagen breakdown and attenuation of lysyl oxidase-mediated collagen cross-linking. SAA synthesized in extravillous trophoblasts may promote their invasiveness into the endometrium in placentation. Here, we summarized the current understanding of SAA in pregnancy with an aim to stimulate in-depth investigation of SAA in pregnancy, which may help better understand how inflammation is initiated in gestational tissues in both normal and abnormal pregnancies.

## 1 Introduction

Inflammation plays a key role in maintaining tissue homeostasis. Both exaggerated or inadequate inflammation can lead to diseases. In pregnancy, intricately-controlled inflammation is required in gestational tissues for implantation, gestational maintenance, and parturition (1–3). Dysregulation of inflammatory reactions in gestational tissues can lead to reproductive disorders, including infertility, spontaneous abortion, preterm birth, pre-eclampsia and gestational diabetes (1–4). Therefore, understanding the regulatory mechanism of inflammatory reactions in gestational tissues may help understand the normal process of pregnancy as well as the pathogenesis of diseases associated with pregnancy. The role of classical inflammatory mediators such as cytokines, chemokines, and prostaglandins in pregnancy has been extensively covered in a bunch of excellent review papers (1–7). However, how inflammation is launched in gestational tissues is still not very well understood.

Damage-associated molecular patterns (DAMPs) are molecules released from damaged or dying cells due to trauma or infection as a component of the innate immune response. DAMPs serve as a warning sign for the organism by launching inflammatory responses (8). Emerging evidence indicates that the acute phase response (APR) protein serum amyloid A (SAA) may function as a DAMP molecule in pregnancy. SAA released into the circulation or synthesized locally in the gestational tissues may help the mother and fetus sense danger signals in pregnancy by launching both specific and non-specific effects in gestational tissues. SAA may be associated with placentation, membrane rupture, parturition, postpartum recovery as well as gestational disorders. Herein, we reviewed the current understanding of SAA in pregnancy by summarizing the available publications in the literature as well as our own studies. To aid in the better understanding of SAA in pregnancy, we will first introduce the general aspects of SAA in the acute phase response briefly.

## 2 SAA in the acute phase response

Our body responds to infection, inflammation, and trauma by implementing APR, an innate body defense response which comprises a range of well-orchestrated physiological and biochemical reactions, to prevent ongoing tissue damage, to destroy the invading organism, and to return the body to normal function (9–11). Central to these reactions is the hepatic production of acute-phase proteins, particularly C-reactive protein (CRP), mannan-binding lectin (MBL) and serum amyloid A (SAA) (11–15). It is known that one of the major functions of CRP and MBL in APR is to act as soluble opsonin-like pattern-recognition receptors so that microorganisms can be tagged for phagocytosis. By contrast, the function of SAA in APR is not entirely understood. However, SAA must be crucial for individual survival because SAA has been conserved across all mammals for several million years (16, 17), and moreover, the plasma levels of SAA is increased by 1000-fold from basal 20-50 μg/mL to 1 mg/mL (80 mM) in APR within 24 hours (18). Over decades of investigations, we are getting to know more about SAA in APR. Several isoforms of SAA with diversified functions and multiple mediating receptors have been identified.

### 2.1 SAA isoforms

**Figure 1:**
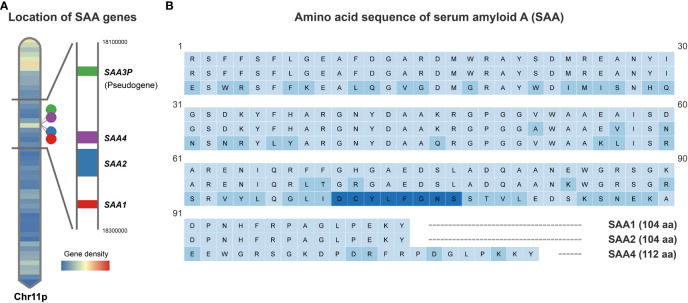
Schematic depiction of human SAA gene locations and protein sequences. **(A)**. Human SAA genes including *SAA1*, *SAA2*, *SAA4* and pseudogene *SAA3P* clustered in a 150-kb region on the short arm of chromosome 11. **(B)**. Comparison of the amino acid sequences encoded by the human *SAA1*, *SAA2* and *SAA4* genes based on UniProt Knowledgebase entries P0DJI8, P0DJI9 and P35542. Amino acid residue variations compared to SAA1 are marked in light blue and an additional 8 amino acids inserted between amino acid residues 69 and 70 of the SAA1/2 in SAA4 are marked in dark blue.

SAA family consists of members coded by different genes which are remarkably conserved across species (13, 16). In humans, there are three SAA-encoding genes (*SAA1, SAA2*, and *SAA4*) and a pseudo gene *SAA3P* which contains an early stop codon (19–21). These four genes are clustered in a 150-kb region on the short arm of chromosome 11 (13, 19) (**Figure 1A**). Of the SAA-encoding genes, *SAA1* and *SAA2* are the acute phase responsive genes which are highly inducible, while *SAA4* is constitutively expressed (13, 20, 22). It is widely accepted that the liver is the primary source of plasma SAA1 and SAA2 in the acute phase response (13, 16, 20), which can be induced by pro-inflammatory cytokines like interleukin-6 (IL-6), tumor necrosis factor-α (TNF-α), interleukin-1β (IL-1β), and interferon-γ (IFN-γ) (13, 23–27). Notably, the stress hormone glucocorticoids are also capable of inducing SAA expression in the liver despite being generally considered as an anti-inflammatory hormone (28). However, it has been shown that glucocorticoids preferentially stimulate the transcription of *SAA1* but not *SAA2* because only *SAA1* promoter harbors a glucocorticoid response element (GRE) (29). The mouse *SAA* genes were mapped to chromosome 7, a region which is analogous to the region of human chromosome 11 (13). Likewise, the expression of *Saa1* and *Saa2* is inducible, while *Saa4* is constitutively expressed in the mouse (13). By contrast, the mouse *Saa3* is not a pseudogene but encodes an inducible SAA3 protein which is believed to be expressed mainly in extrahepatic tissues (13). Instead, *Saa5* is a pseudogene in the mouse (13).

The human SAA1 and 2 are small proteins consisting of 104 amino acids and share 92% of amino acids, while SAA4 carries an additional 8 amino acids inserted between amino acids 69 and 70 of the inducible SAAs (13, 20) (**Figure 1B**). The N-terminal of SAA harbors a hydrophobic α-helical domain, while the C-terminal is essential for the maintenance of SAA structure (13, 17). Proteolytic cleavage of the C-terminal results in the formation of a 76 amino acid N-terminal fragment, which is subject to aggregation and formation of highly-ordered β-sheets, a structure commonly seen in microfibrils of the amyloid deposit (13, 17, 30).

### 2.2 Functions of SAA in APR

The SAA molecule is amphipathic, but mostly lipophilic in circulation (31). Like the transportation of other lipophilic hormones such as the steroid and thyroid hormones, SAA is also transported by a carrier in the blood (31). The water-insoluble N-terminal α-helical domain of SAA carries a binding site for the high-density lipoprotein (HDL). It has been estimated that 95% of SAA released from the liver is transported in HDL despite that a small fraction of lipid-free form (about 50 μg/mL) also exists in the circulation, which may be in equilibrium with the fraction partitioned in HDL (31–33). Since HDL binding sequesters SAA from its biological activities, HDL is considered as a natural inhibitor of SAA biological activities (13, 34, 35). In addition, capture of SAA displaces the original apolipoproteins (Apo-AI) and cholesterol in HDL, and this alteration in HDL composition will increase the affinity of HDL for macrophages (36). It is known that macrophages are engaged in the clearance of dead cells at the injury site, which may overload the macrophages with cholesterol from the engulfed cell debris. Studies have shown that capture of SAA not only increases the affinity of HDL for macrophages, but also facilitates cholesterol efflux from the cholesterol-loaded macrophages to HDL. By this way, the phagocytic activity of macrophages can be restored, and the evacuated cholesterol can be recycled to be used by newly regenerated cells for the repairment damaged tissues (16, 37, 38). Therefore, it is suggested that increased proportion of SAA-containing HDL in the circulation in APR is to ensure individual survival at the early stage.

In addition to the liver-derived SAA, cells in the inflamed tissue including immune cells, fibroblasts, endothelial cells, epithelial cells, adipocytes, and smooth muscle cells are also capable of producing SAA (22, 39–44), which may contribute to a pool of lipid-free SAA together with the liver-derived SAA in the local tissue. This pool of free SAA has been shown to participate in immunomodulation, immunosurveillance and tissue remodeling in the inflamed tissue. SAA has been shown to act as a humoral DAMP molecule to enhance the chemotaxis of neutrophils, monocytes, mast cells, and T lymphocytes, as well as the production of a wide array of inflammatory mediators, including pro-inflammatory cytokines, prostaglandins and matrix metalloproteinases (MMPs) (13, 40, 41, 45–54). In addition, SAA has also been shown to induce macrophage polarization toward both pro-inflammatory M1 phenotype and anti-inflammatory M2 phenotype (55–57). The M2 phenotype helps resolve inflammation by secreting anti-inflammatory cytokines and clearing dead cell debris. Given all these properties of SAA in the inflamed tissue, it is likely that SAA helps the body survive the traumatic insults through reversible homeostatic inflammatory reactions by functioning as an early responsive host-derived DAMP molecule.

### 2.3 SAA receptors

The apparent importance of SAA in APR has aroused great interest in the identification of its mediating receptors, but the delineation develops slowly. To date, several cell surface receptors have been identified, including the G protein-coupled FPRL1 (formyl peptide receptor like–1) receptor, the pattern recognition receptors RAGE (receptor for advanced glycation end products) and TLR2/4 (toll-like receptor 2/4), and the scavenger receptors CD36 (cluster of differentiation 36) and CLA-1 (CD36 and LIMPII Analogous-1) (35, 54, 58–64). Activation of FPRL1, RAGE and TLR2/4 has been shown to be responsible for SAA-induced inflammatory reactions including macrophage activation, chemotaxis, cytokine expression, MMP9 and nitric oxide production with the activation of ERK and p38 MAPK and NF-κB pathways (35, 54, 58–61, 63). The scavenger receptor CLA-1 and CD36 have been demonstrated to mediate the effect of HDL-SAA on cholesterol efflux from cells (64, 65) and SAA-induced pro-inflammatory cytokine secretion (62) respectively. Although multiple SAA receptors have been identified, it is unclear whether these receptors are expressed in a cell-specific manner or multiple SAA receptors are jointly expressed in the same cell.

In summary, there are three SAA isoforms in humans, and the predominant role of the inducible SAAs in APR may help the body overcome traumatic insults by launching reversible homeostatic inflammatory reactions as an early host-derived DAMP molecule. For a comprehensive description of SAA in APR, the reader is referred to a wealth of elegant reviews in this area (13, 16, 20, 22, 52). Here, in the subsequent sections, we will focus on SAA in pregnancy, a subject that has not received adequate attention.

## 3 SAA in pregnancy

Inflammation is implicated in both normal and abnormal pregnancies (1–4). While optimal inflammation in gestational tissues is an indispensable event of implantation and parturition processes, aberrant inflammation is associated with pregnancy disorders such as abortion, premature preterm rupture of membranes (PPROM), preterm birth, gestational diabetes (GDM) and pre-eclampsia (66–69). In view of the role of SAA in inflammation in APR and the inflammatory states of normal and abnormal pregnancies, investigators have been trying to disclose the role of SAA in pregnancy for some time. Evidence gathered so far indicates that SAA in maternal circulation may function as a non-specific DAMP molecule in response to tissue inflammation and damage incurred in parturition and gestational disorders. However, SAA synthesized locally in gestational tissues may function as an early host-derived DAMP molecule exerting more specific effects in placentation, membrane rupture and parturition, thereby helping the embryo implant into the endometrium and aiding the fetus to escape from the endangered *in-utero* environment.

### 3.1 Changes of maternal plasma SAA in normal pregnancy

In view of the inflammatory effects of SAA and the dramatic hormonal changes in pregnancy, earlier studies assumed that maternal plasma SAA levels in normal pregnancy should differ from that in non-pregnancy. Ostensen et al. measured SAA levels in the serial plasma samples collected from the same pregnant woman, and found that maternal plasma SAA levels remained unaltered in the first, second and third trimesters, but manifested a dramatic increase immediately after parturition, which resumed normalization within the first 2 weeks postpartum (70). Subsequent studies confirmed the findings by Ostensen et al. (71, 72). These later studies established that maternal plasma SAA levels stayed unaltered during pregnancy but began to rise immediately after parturition and reached a plateau at 24 hours postpartum (71, 72). Of interest, these studies also revealed that SAA concentrations in the maternal blood were much higher than that in umbilical cord blood (71, 72), indicating that there is a lack of transplacental transfer of SAA, and any increases in SAA levels in cord blood may reflect increased fetal production in conditions such as fetal infection or trauma. However, only a few studies addressed whether SAA has already manifested a change at the onset of labor. Cicarelli et al. found that some but not all of the cases they examined displayed a rise of SAA levels in maternal circulation at the moment of delivery (72). By measuring SAA levels in the serial maternal venous blood samples collected before (1–2 days before labor onset) and after onset of labor as well as 24 hours after delivery, we found that there was a significant increase in SAA1 levels in maternal circulation after onset of labor, but this increase was much less than the increase 24 hours after delivery (40). It remains unsettled whether this increase after onset of labor is part of the initiating mechanism of parturition or a mere non-specific reflection of the inflammatory state of gestational tissues at parturition. Nonetheless, all these studies including ours showed a marked increase in maternal blood SAA levels at postpartum. It is very likely that this postpartum SAA elevation in maternal circulation is part of the non-specific acute phase response of the body to tissue inflammation and damage experienced in parturition (70), which may help the mother enhance host defense and repair the tissue damage.

### 3.2 Changes of maternal plasma SAA in gestational disorders

#### 3.2.1 PPROM and chorioamnionitis

Fetal membranes are consisted of amnion and chorion layers, which enclose the amniotic fluid, and function as a barrier to ascending infections for the protection of the fetus. Preterm premature rupture of membranes (PPROM) occurs in 8–10% of all pregnancies (4, 73). Ruptured membranes are exposed to ascending infections with a high likelihood of the development of chorioamnionitis. Chorioamnionitis is a major cause of preterm birth, and unrestrained chorioamnionitis can spread infection to both mother and fetus leading to deadly outcomes (4, 74). It is estimated that PPROM accounts for approximately one-third of preterm birth and 18–20% of perinatal deaths (5, 6, 9). Therefore, understanding the cause of PPROM and chorioamnionitis is of utmost importance for the prevention of preterm birth as well as for the safety of both mother and fetus. In view of the tissue damage incurred and the high risk of chorioamnionitis in PPROM, SAA was expected to rise in PPROM, particularly those with infection. Yang et al. found that SAA 1 and 2 were among the proteins which were significantly elevated in maternal blood in lipopolysaccharide (LPS)-induced preterm birth in the mouse (75). In human studies, Koseoglu et al. found that SAA levels in maternal blood were significantly elevated in the PPROM group compared to the control group (76), and Kayabas et al. demonstrated that SAA levels in maternal circulation were further increased in patients with clinical chorioamnionitis (107 µg/mL) when compared with patients with only PPROM but not chorioamnionitis (21 µg/mL) (77). We also found that SAA1 levels were markedly increased in maternal circulation in preterm birth with chorioamnionitis when compared with gestational age-matched iatrogenic preterm birth (40). In addition to the rise of SAA levels in maternal circulation, SAA levels in cord blood were also significantly higher in patients with PPROM (115 µg/mL) than in patients without PPROM (26 µg/mL) (77). These results suggest that both mother and fetus may respond to PPROM, and the response may be particularly strong when chorioamnionitis is present. However, the exact role of increased SAA levels in PPROM and chorioamnionitis is not well understood. It is likely that increased SAA levels in maternal circulation in PPROM and chorioamnionitis are the acute phase response of the body to the mediators released from damaged and infected membranes.

#### 3.2.2 Pre-eclampsia and placenta accreta

Pre-eclampsia is a hypertensive disease that develops during pregnancy, which accounts for 2% to 8% of pregnancy-related complications (78). The exact cause for pre-eclampsia remains elusive. Apart from majorly insufficient blood flow to the placenta due to shallow invasion of extravillous trophoblasts (EVT) into the endometrium (79), vascular endothelial dysfunction associated with maternal systemic inflammation has also been suggested to account for the pathogenesis of pre-eclampsia (68, 69, 80). It has been shown that maternal plasma SAA levels were significantly increased in pre-eclampsia, and were further increased in eclampsia and HELLP (hemolysis, elevated liver enzymes, low platelet count) syndrome (81–83). However, there is also a study that failed to show any changes of SAA levels in patients with pre-eclampsia irrespective of severity (84). It is not known what causes the disparity. The early or late onset of the disease may affect the results. Although these studies described that the recruited patients were diagnosed pre-eclampsia after 20 weeks’ gestation, they did not mention when patients develop hypertension.

In contrast to the shallow invasiveness of EVTs in pre-eclampsia, excessive invasion of EVTs can result in placenta accreta, which may lead to deadly bleeding at parturition. Zakaria et al. reported that SAA levels in maternal blood were significantly increased in patients with placenta accreta (85). As SAA levels were elevated in situations of both shallow and excessive invasiveness of EVTs, it is likely that the alteration of SAA levels in maternal blood in pre-eclampsia and placenta accreta are the non-specific reflection of the inflammatory and injury states of the diseases.

#### 3.2.3 Gestational diabetes

The prevalence of gestational diabetes mellitus (GDM) is about 3 to 15% of pregnancies, which is thought to arise from adaptive failures to pregnancy with maternal obesity as an important contributor (66, 86). Pregnancies complicated with GDM are more likely to develop maternal and fetal complications, such as hypertensive disorders, pre-eclampsia, preterm birth, and increased risk of developing diabetes in later lives. Accumulating evidence indicates that both obesity and GDM may be associated with a state of low-grade chronic inflammation (66, 67). As a sensitive marker of inflammatory diseases, SAA is hypothesized to contribute to the inflammatory state of patients with GDM. Erin et al. found that maternal circulating SAA levels were significantly higher in women with GDM when compared with healthy pregnant controls (87). Their correlation analysis showed that SAA levels were significantly correlated with age, BMI, mean arterial blood pressure, glucose tolerance and carotid intima-media thickness (CIMT) (87), a valid predictor of atherosclerosis. Thus, they suggested that increased maternal plasma SAA levels in GDM might be an indicator of an increased risk of subclinical atherosclerosis and future atherosclerotic heart disease (87). In contrast to the findings by Erin et al., Pöyhönen-Alho et al. showed that both SAA and CRP levels (SAA: 1.24 ± 0.27 mg/L, CRP: 0.60 ± 0.37 mg/L) were reluctant to change in pregnant women with GDM when compared with normal pregnant women (SAA: 1.32 ± 0.38 mg/L, CRP: 0.59 ± 0.61 mg/L) (88). Notably, their CRP data were also in contradiction with previous studies which showed elevated CRP levels in GDM (89, 90). Further investigations may be required to affirm the change of maternal blood SAA levels and its significance in GDM.

#### 3.2.4 Recurrent spontaneous abortion

Recurrent spontaneous abortion (RSA) is defined as three or more consecutive spontaneous pregnancy losses (91), which occurs in approximately 1% of pregnancies (92). However, about half of the RSA cases are unexplained pregnancy losses (93, 94). Ibrahim et al. demonstrated that plasma SAA levels were significantly increased in women with unexplained RSA (Median 50.0 μg/mL, interquartile range 26.0-69.0) when compared with the control group (Median 11.6 μg/mL, interquartile range 6.2-15.5; P<0.001) (95). These findings were confirmed by an independent group (96). However, the significance of SAA elevation in RSA was not investigated, but it was suggested that SAA might be a biomarker of RSA (95).

The reported changes of SAA levels in the circulation in different gestational disorders are summarized in **Table 1**. It appears that SAA levels in maternal circulation may not be an ideal biomarker for specific pregnancy complications, but the extent of SAA elevation may reflect the severity of gestational disorders.

**Table 1:** SAA levels in the circulation in gestational disorders.

Gestational disorders	Sample	SAA concentration		Statistics	Authors	References
		Disorder group	Control group			
PPROM	Maternal serum	905.16 ± 2652.79 ng/mL	78.71 ± 100.09 ng/mL	Mean ± SD	Koseoglu et al.	(76)
	Maternal serum	80 ± 44 μg/mL	10 ± 7.2 μg/mL	Mean ± SD	Kayabas et al.	(77)
	Umbilical cord serum	84 ± 40 μg/mL	7.4 ± 3.0 μg/mL	Mean ± SD		
Chorioamnionitis	Maternal serum	107 ± 8.4 μg/mL	21 ± 4.5 μg/mL	Mean ± SD	Kayabas et al.	(77)
	Umbilical cord serum	115 ± 12 μg/mL	26 ± 32 μg/mL	Mean ± SD		
	Maternal serum	25.88 ± 5.91 μg/mL	1.48 ± 1.52 μg/mL	Mean ± SEM	Gan et al.*	(40)
Pre-eclampsia	Maternal serum	28.2 (7.2-135) ng/L	7.8 (4.65-24.6) ng/L	Mean (Min-Max)	Engin-Ustun et al.	(81)
	Maternal serum	9.0 ± 3 mg/mL	4.7 ± 2.6 mg/mL	Mean ± SD	Uckan et al.	(82)
	Maternal plasma	3.94 (1.05-33.00) mg/L	4.31 (0.95–24.98) mg/L	Median (2.5-97.5^th^ percentiles)	Kristensen et al.	(84)
HELLP	Maternal serum	12.1 ± 1 mg/mL	4.7 ± 2.6 mg/mL	Mean ± SD	Uckan et al.	(82)
	Maternal plasma	7.41 (3.57–10.26) mg/L	2.28 (1.68–3.09) mg/L	Mean (Min-Max)	Heitner et al.	(83)
Eclampsia	Maternal serum	12.2 ± 0.4 mg/mL	4.7 ± 2.6 mg/mL	Mean ± SD	Uckan et al.	(82)
Placenta accreta	Maternal serum	19.86 ± 5.72 μg/mL	11.56 ± 2.19 μg/mL	Mean ± SD	Zakaria et al.	(85)
Gestational diabetes	Maternal serum	531.7 ± 91.7 ng/mL	465.6 ± 77.6 ng/mL	Mean ± SD	Eren et al.	(87)
	Maternal plasma	1.24 ± 0.27 mg/L	1.32 ± 0.38 mg/L	Mean ± SD	Poyhonen-Alho et al.	(88)
Recurrent spontaneous abortion	Maternal serum	50.0 (26.0-69.0) μg/mL	11.6 (6.2-15.5) μg/mL ^#^	Median (25-75^th^ percentiles)	Ibrahim et al.	(95)
	Maternal serum	32.92 ± 14.45 μg/mL	15.89 ± 6.12 μg/mL	Mean ± SD	Ming et al.	(96)

*SAA1 was measured specifically; # Spontaneous abortion but not diagnosed with RSA.

## 4 SAA synthesized within gestational tissues

It is widely accepted that the liver is the major source of acute phase SAA in the circulation. However, accumulating evidence indicates that a wide range of cells in extrahepatic tissues including immune cells, fibroblasts, endothelial cells, epithelial cells, adipocytes, and smooth muscle cells are capable of *de novo* synthesis of SAA (22, 39–44). SAA produced locally in extrahepatic tissues may be more efficient than the liver-derived SAA in launching inflammatory reactions within the tissue. In pregnancy, almost all gestational tissues including the placenta, fetal membranes and uterus are capable of SAA synthesis, and SAA synthesized in these gestational tissues has been shown to be implicated in the initiation of inflammatory processes as well as specific actions involved in placentation, membrane rupture and parturition.

### 4.1 Placenta

The placenta is a temporary fetal organ in pregnancy, which plays critical roles in nutrient, gas, and waste exchange between maternal and fetal circulations (97, 98). The placental cytotrophoblasts can either differentiate into villous syncytiotrophoblasts or EVTs. The villous syncytiotrophoblasts serve as an important endocrine organ producing critical hormones which not only maintain pregnancy, but also promote parturition. In contrast to the villous trophoblasts, EVTs invade into the endometrium whereby they remodel uterine spiral arteries for the establishment of implantation (99). Sandri et al. demonstrated that SAA was expressed in EVTs and decidual cells at the maternal-fetal interface in humans (100), where SAA promoted the invasion of EVTs into the endometrium through toll-like receptor 4 (TLR4)-mediated induction of MMP2 and 9 (100). This function of SAA in EVTs mimics very much the situation of tumor cells in metastasis. Both circulatory and local SAA levels have been shown to be increased in many tumors (101, 102), and SAA has been shown to aid in tumor cell invasion and metastasis by enhancing ECM degradation through induction of MMPs (49, 58, 103).

In addition to EVTs, we and others demonstrated that SAA was also synthesized in human placental villous trophoblasts (40, 100, 104, 105), and its expression was significantly increased upon syncytialization of trophoblasts (40). However, it appeared that SAA played no role in the syncytialization of villous trophoblasts. SAA treatment of BeWo cells (a trophoblast cell line) did not significantly increase the number of multinucleated syncytiotrophoblasts as observed with F-actin staining, and instead, SAA inhibited the secretion of hCG, a hormone synthesized primarily by syncytiotrophoblasts, in BeWo cells (100). However, SAA may participate in parturition by launching inflammatory reactions through sensing the inflammatory stimuli as well as by executing specific actions within gestational tissues.

As previously described, inflammation of gestational tissues is indispensable in parturition (1, 3, 106). Depending on the presence or absence of infection, inflammation of the gestational tissues can be classified into infectious and sterile inflammation (1, 3, 7, 106, 107). While infectious inflammation plays an important role in infection-induced preterm birth, sterile inflammation is more crucial in normal parturition. Although oxidative stress and cell senescence have been suggested to play a role in the initiation of sterile inflammation in parturition (7, 108), it remains elusive whether there exists any humoral factor involved in the initiation of sterile inflammation in gestational tissues. We found that SAA1 expression was significantly increased in the placenta villous tissue in normal spontaneous deliveries without infection at term compared with elective c section without labor at term (40). SAA1 in maternal blood was also significantly increased in infection-induced preterm birth compared with iatrogenic preterm birth without labor and histologic chorioamnionitis (40). Further investigation showed that inflammatory stimuli such as LPS and pro-inflammatory cytokines could stimulate the expression of SAA1 in placenta villous trophoblasts, and in turn, SAA1 increased the expression of inflammatory mediators including IL-1β, IL-8, TNF-α and cyclooxygenase 2 (COX-2) with concomitantly increased prostaglandin F2α (PGF2α) production (40), a major prostaglandin isoform synthesized by the placenta (109). Prostaglandins E2 (PGE2) and PGF2α are regarded as the final common mediators of labor onset in virtually all species, and they are not only potent stimulators of myometrial contraction, but also strong inducers of cervical ripening (109). Given the crucial role of prostaglandins and inflammation in parturition, we believe that SAA1 in the placenta is implicated in the creation of an inflammatory environment resulting in the increased prostaglandin production for parturition. Studies in the mouse support such a notion (40). SAA1 was found in the junctional zone of the mouse placenta, which was significantly increased by LPS administration. Moreover, intraperitoneal injection of SAA1 (8 μg/kg body weight) on gestational day 16.5 induced preterm birth with concurrently increased abundance of IL-1β, TNF-α, and COX-2 in the mouse placenta (40). These data gathered in the placenta suggest that SAA may be a humoral factor involved in the initiation of both sterile and infectious inflammation in gestational tissues in parturition.

In addition to inflammatory mediators, the stress hormone glucocorticoids also stimulated SAA1 expression in placental villous trophoblasts (40). The findings in the placenta are in line with the findings in the liver where glucocorticoids have been shown to stimulate SAA1 expression either on their own or in synergy with pro-inflammatory cytokines (24, 28). It is widely accepted that glucocorticoids play an important role in the initiation of parturition across different species including humans (110–114). Despite the differences in their origins and target issues, induction of COX-2 expression with concomitantly increased PGE2 and PGF2α production comprises one of the key effects of glucocorticoids in parturition in virtually all species (115–119). The finding that glucocorticoids stimulated the expression of SAA1 in human placental villous trophoblasts indicates that induction of SAA1 expression in gestational tissues may also play a role in glucocorticoid-induced parturition. In addition to the induction of SAA expression in the placenta, glucocorticoids also induce SAA expression either on their own or in synergy with SAA in the fetal membranes, which will be described in the following section.

### 4.2 Fetal membranes

As previously described, the fetal membranes make up the amniotic sac which provides protection for the fetus (120). Of the amnion and chorion layers, the amnion is known to be more tensile (114, 120–122). Toward the end of gestation, the amnion layer normally undergoes extensive extracellular matrix (ECM) remodeling as well as cell apoptosis and senescence in order to rupture in parturition (7, 108, 121, 122). However, premature preterm rupture of membranes may predispose the membranes to ascending infection resulting in the development of chorioamnionitis and preterm birth. In addition to the protective role described above, the fetal membranes are also known as a rich source of vital biochemicals which plays a pivotal role in parturition (108, 109, 114). Among these biochemicals, PGE2 and cortisol should be emphasized particularly (109). The fetal membranes not only produce the most PGE2 in pregnancy (109, 123, 124) but also possess the highest capacity of cortisol regeneration through 11β-hydroxysteroid dehydrogenase 1 (11β-HSD1) among fetal tissues (114, 125–127). Moreover, both PGE2 and cortisol can stimulate the expression of COX-2 and 11β-HSD1, the enzymes responsible for their respective production in the fetal membranes (115, 117–119, 128–131), thus forming a feedforward loop of mutual enhancement between cortisol and PGE2 production, which is believed to be a crucial feedforward mechanism underlying membrane activation at parturition (114, 125).

**Figure 2:**
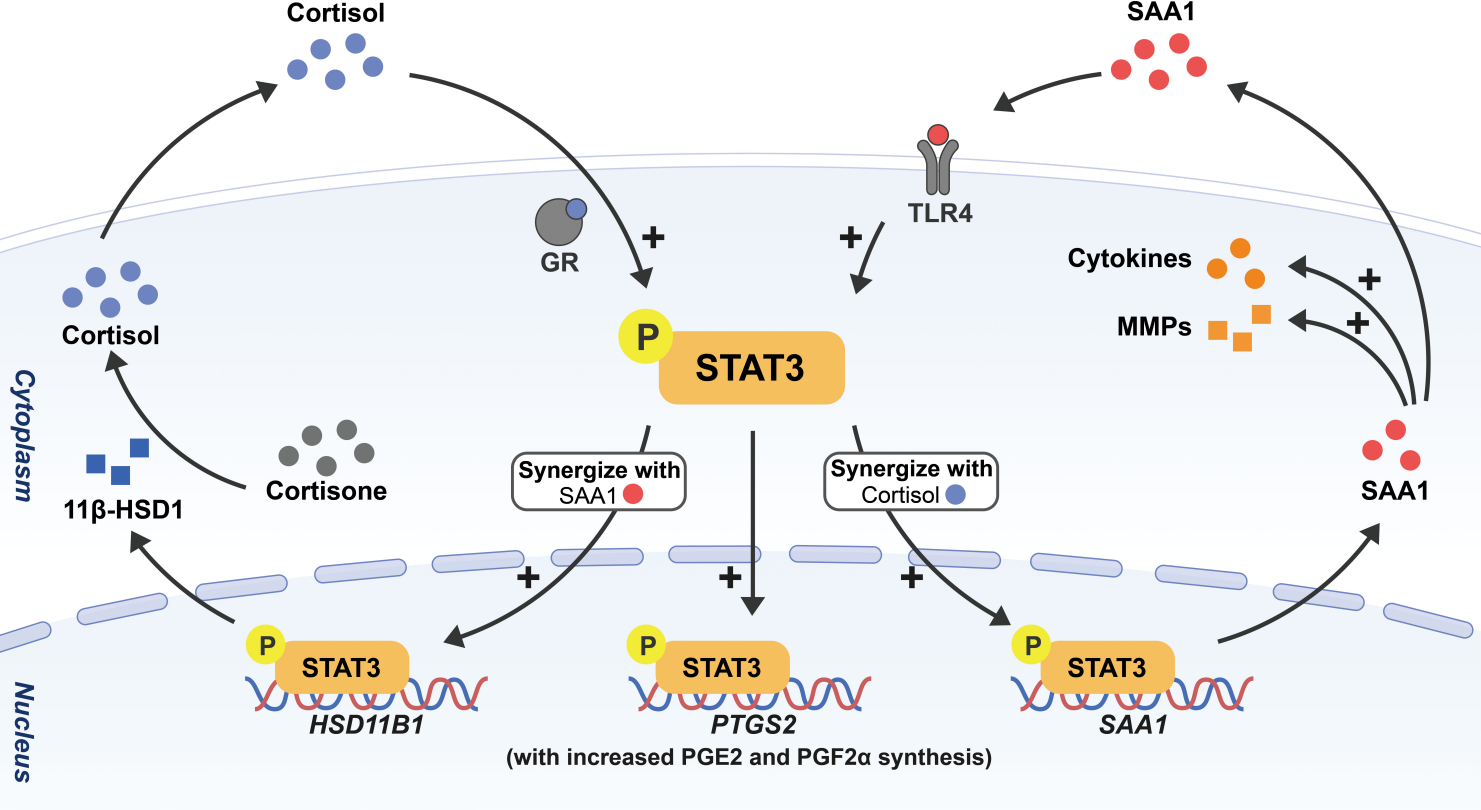
Schematic drawing depicting the feedforward loop among SAA, cortisol and prostaglandins in the activation of fetal membranes with STAT3 as a central transcription factor. Through phosphorylation of STAT3, cortisol and SAA1 can induce the expression of not only 11β-HSD1 but also SAA1 *per se* synergistically in amnion fibroblasts, and in turn, 11β-HSD1 converts biologically inactive cortisone to cortisol. Cortisol and SAA1 further stimulate the expression of COX-2 (encoded by *PTGS2*) with subsequent increased PGE2 and PGF2α synthesis. In addition, SAA1 also increases the expression of a number of pro-inflammatory cytokines and MMPs in amnion fibroblasts. All these effects will lead to membrane activation and ECM remodeling in parturition.

In addition to the identified biochemicals, we discovered that almost all cell types including amnion fibroblasts, epithelial cells and chorion trophoblasts in human fetal membranes are capable of *de novo* synthesis of SAA1 (41). Interestingly, by using cultured human amnion fibroblasts, a major site of cortisol regeneration and PGE2 production, we demonstrated that cortisol and SAA1 synergistically induced the expression of not only 11β-HSD1 but also SAA1 *per se* (132, 133), and in turn, SAA1 produced by fibroblasts might induce the expression of a number of inflammatory mediators including IL-1β, IL-6 as well as COX-2 with increased PGE2 production in fibroblasts in an autocrine or paracrine manner (41). Our data suggest that SAA1 may be part of the feedforward loop described above in membrane activation at parturition. Further investigation revealed that the synergistic induction of 11β-HSD1 and SAA1 expression by cortisol and SAA1 involved the participation of the transcription factor STAT3 in amnion fibroblasts (132). Of interest, STAT3 has been shown to mediate the transcription of several acute phase genes including SAA1 in the liver (134, 135). Previously, we also demonstrated that STAT3 was a key transcription factor mediating the induction of COX-2 expression by glucocorticoids in amnion fibroblasts (119). Given all these transcriptive effects in amnion fibroblasts, STAT3 appears to be a central transcription factor orchestrating the expression of multiple biochemicals of the feedforward loop in the activation of membranes at parturition (**Figure 2**).

In preparation for membrane rupture, the amnion undergoes profound ECM remodeling. Interestingly, SAA synthesized locally in synovial cells was found to be a stimulator of the expression of collagenase and other MMPs, which might be responsible for joint destruction in rheumatism (51, 136, 137). We found that SAA1 was also involved in ECM remodeling in the amnion by inducing the expression of several MMPs including MMP-1, MMP-2, MMP-8, MMP-9 and MMP-13 (138, 139), and decreasing the expression of lysyl oxidase‐like 1 (LOXL1) (139), a collagen cross-linking enzyme, in amnion fibroblasts. In addition to the enhancement of enzymatic cleavage of collagens by MMPs, we also found that SAA1 induced autophagy-mediated collagen breakdown in amnion fibroblasts (138). These findings highlight that upon sensing danger signals, increased SAA synthesis locally in the amnion may participate in parturition by launching the feedforward loop of membrane activation as well as membrane rupture through multiple ECM remodeling approaches.

### 4.3 Uterus

It is within the uterus that the conception is established and the fetus develops. A successful implantation of the fertilized egg requires the proper timing of decidualization of the endometrium (140). Impairment of this process can lead to a series of pregnancy disorders (140–142). Recently, Goolam et al. demonstrated that *SAA3* is robustly upregulated (272-fold) in the decidua of a mouse model with defective formation of primary decidual zone (143). In view of the role of SAA in the invasiveness of EVTs and in the inflammatory reactions of gestational tissues (40, 41, 100, 132, 139), it is possible that SAA synthesized locally at the fetal-maternal interface plays a crucial role in the fine regulation of inflammatory reactions and tissue remodeling for the establishment of pregnancy.

In addition to the endometrium, the myometrium also undergoes dramatic changes during pregnancy. The myometrium is distended and quiet during gestation, but becomes highly contractile in parturition. It has been suggested that inflammation may be involved in the myometrium transition from a quiescent to a contractile phenotype (6, 144). Jiang et al. demonstrated that SAA1 expression was significantly increased in human myometrium at term laboring compared to non-laboring (145), and they found that SAA1 stimulated the expression of pro-inflammatory cytokines (IL-8, IL-6), chemokines (CXCL5, CCL2), adhesion molecules (ICAM1, ICAM5) and PGE2 *via* activating the Yes-associated protein (YAP) pathway in human primary myometrial cells (145). These results suggest that SAA1 synthesized in the myometrium may help create an inflammatory microenvironment in the myometrium to assist its transition to the contractile phenotype in parturition.

Notably, almost all the inflammatory, endocrine and ECM remodeling effects of SAA1 in gestational tissues were mediated by the toll-like receptor TLR4 (40, 41, 100, 132, 138, 139). The toll-like receptors function as pattern recognition receptors (PRRs) in the detection of danger signals including a wide range of microbial products and host-derived DAMP proteins (146, 147). High-mobility group box 1 (HMGB1) is such a host-derived protein that interacts with multiple TLRs (148). Given the inflammatory properties of SAA depicted above, we believe that SAA may be another host-derived DAMP molecule which can be sensed by TLR4 in gestational tissues to initiate tissue-controlled inflammation and tissue remodeling at parturition. The idea is reinforced by the findings that the abundance of SAA1 was significantly increased in gestational tissues in parturition with or without infection, and upon stimulation by bacterial products, pro-inflammatory cytokines and stress hormone glucocorticoids (40, 41, 132), and in turn, SAA stimulated the production of pro-inflammatory mediators including SAA *per se*, tissue remodeling proteases MMPs as well as prostaglandins PGE2 and PGF2α, the common mediators of labor onset, in gestational tissues (40, 41, 100, 132, 138, 139).

## 5 Conclusions and perspectives

**Figure 3:**
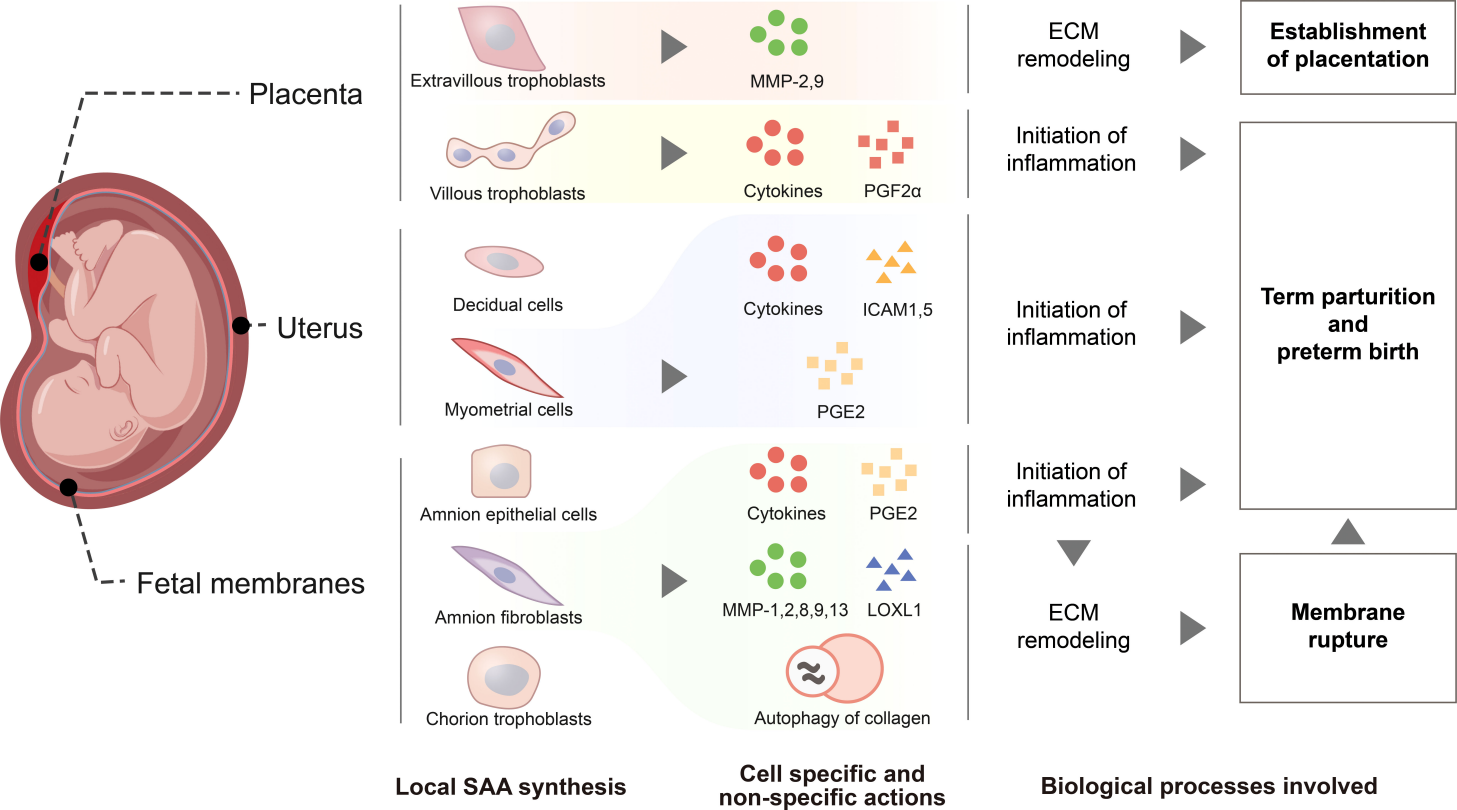
Schematic drawing depicting functions of SAA synthesized within gestational tissues. *De novo* SAA synthesis is detected in extravillous trophoblasts and syncytiotrophoblasts of the placenta, myometrial and decidual cells of the uterus, epithelial and fibroblast cells of the amnion, cytotrophoblasts of the chorionic membrane. In extravillous trophoblasts, SAA may promote their invasion into the endometrium through induction of metalloproteases 2 and 9 for the establishment of placentation. SAA synthesized within myometrium, placenta and fetal membranes may promote parturition *via* enhancing inflammatory reactions through induction of pro-inflammatory cytokines and other inflammatory mediators including prostaglandins PGF2α and PGE2, ICAM1 and ICAM5, the adhesion molecules for the infiltrated leukocytes. Moreover, SAA may also facilitate fetal membrane rupture in parturition through induction of extracellular matrix remodeling *via* increasing the expression of metalloproteases 1, 2, 8, 9 and 13, decreasing the expression of lysyl oxidase‐like 1 and inducing autophagy-mediated collagen breakdown.

SAA is emerging as a host-derived DAMP molecule in the detection of either sterile or infectious signals in the initiation of inflammation in gestational tissues. SAA undergoes a modest rise during parturition but a dramatic rise in postpartum in maternal circulation, which may be a non-specific host innate immune response to the inflammation and trauma experienced during parturition. In gestational disorders such as PPROM, infection-induced preterm birth, pre-eclampsia, gestational diabetes and recurrent spontaneous abortion, maternal plasma SAA levels may increase non-specifically during pregnancy possibly due to the inflammatory nature of these diseases. Thus, SAA levels in maternal circulation may not be an ideal biomarker for specific pregnancy complications, but the extent of SAA elevation may reflect the severity of gestational disorders. Although the liver has been known to be a major source of plasma SAA in APR, cells in gestational tissues including villous and extravillous trophoblasts of the placenta, amnion epithelial and fibroblast cells, and chorion trophoblasts of the fetal membranes, decidual and myometrial cells of the uterus are all capable of SAA1 synthesis. SAA produced locally in gestational tissues may function as a TLR4-sensed DAMP molecule to be involved in the inflammatory reactions as well as specific actions associated with placentation, membrane rupture and initiation of parturition (**Figure 3**). However, the role of SAA in pregnancy is just starting to emerge. Several unresolved issues await further investigation. It is intriguing why our body needs two inducible SAAs and a constituent SAA4. It is necessary to decipher whether these isoforms play any differential roles in pregnancy in the future. Moreover, thorough investigations are required to understand the exact role of SAA in normal pregnancy, particularly in postpartum and gestational disorders. We believe that in-depth investigation of SAA in pregnancy may help better understand how inflammation is initiated in gestational tissues in both normal and abnormal pregnancies.

## Author contributions

All authors listed have made a substantial, direct, and intellectual contribution to the work and approved it for publication.

## Funding

This work was supported by National Natural Science Foundation of China (81830042 and 82071677), National Key R & D Program of China (2020YFA0803900) and Innovative Research Team of High-level Local Universities in Shanghai (SHSMU-ZLCX20210200).

## Conflict of interest

The authors declare that the research was conducted in the absence of any commercial or financial relationships that could be construed as a potential conflict of interest.

## Publisher’s note

All claims expressed in this article are solely those of the authors and do not necessarily represent those of their affiliated organizations, or those of the publisher, the editors and the reviewers. Any product that may be evaluated in this article, or claim that may be made by its manufacturer, is not guaranteed or endorsed by the publisher.
